# Morphological and genetic variability in cosmopolitan tardigrade species - *Paramacrobiotus fairbanksi* Schill, Förster, Dandekar & Wolf, 2010

**DOI:** 10.21203/rs.3.rs-2736709/v2

**Published:** 2023-07-17

**Authors:** Pushpalata Kayastha, Wiktoria Szydło, Monika Mioduchowska, Łukasz Kaczmarek

**Affiliations:** 1 Department of Animal Taxonomy and Ecology, Faculty of Biology, Adam Mickiewicz University, Poznań, Uniwersytetu Poznańskiego 6, 61-614 Poznań, Poland;; 2 Center for Advanced Technology, Adam Mickiewicz University in Poznań, Uniwersytetu Poznańskiego 10, 61-614 Poznań, Poland;; 3 Population Ecology Lab, Faculty of Biology, Adam Mickiewicz University, Poznań, Poland; 4 Department of Evolutionary Genetics and Biosystematics, Faculty of Biology, University of Gdańsk, Poland;

**Keywords:** cosmopolitism, dispersal, ‘Everything is Everywhere’ hypothesis, Tardigrada, water bears, zoogeography

## Abstract

*Paramacrobiotus fairbanksi* was described from Alaska (USA) based on integrative taxonomy and later reported from various geographical locations making it a true cosmopolitan species. The ‘Everything is Everywhere’ (EiE) hypothesis assumes that microscopic organisms have unique features that help them to inhabit many different environments, meaning they can be considered cosmopolitan. In the present work we report four new populations of *Pam. fairbanksi* from the Northern Hemisphere which suggests that the ‘EiE’ hypothesis is true, at least for some tardigrade species. We also compared all known populations of *Pam. fairbanksi* at the genetic and morphological levels. The p-distances between COI haplotypes of all sequenced *Pam. fairbanksi* populations from Albania, Antarctica, Canada, Italy, Madeira, Mongolia, Spain, USA and Poland ranged from 0.002% to 0.005%. In total, twelve haplotypes (H1-H12) of COI gene fragments were identified. We also report statistically significant morphometrical differences of species even though they were cultured and bred in the same laboratory conditions, and propose epigenetic factor as a main cause rather than temperature, predation risk and food availability. Furthermore, we also discuss differences in the potential distribution of two *Paramacrobiotus* species.

## Introduction

The Phylum Tardigrada currently consists of *ca*. 1,500 species^1^ that inhabit terrestrial and aquatic environments throughout the world^2^. Currently there are 33 families, 159 genera, 1464 species and 21 additional subspecies within this phylum^1^. *Paramacrobiotus fairbanksi* Schill, Förster, Dandekar & Wolf, 2010^3^ was described from Alaska (USA) and reported from the Antarctic, Italy, Poland and Spain^4^ (reported as *Macrobiotus richtersi* Murray, 1911^5^)^6,7,8,9^. It is a large-size (up to 800 μm) parthenogenetic *Paramacrobiotus* found mostly in mosses and can be shortly characterized by white or transparent cuticle without pores, three bands of teeth in the oral cavity, three macroplacoids and microplacoid in pharynx (*richtersi* group), smooth lunules under all claws, granulation on all legs, and eggs with reticulated conical processes without caps or spines. *Pam. fairbanksi* is a triploid species^8^ inhabiting various locations throughout the globe. The species is an omnivore, i.e., it feeds on algae, cyanobacteria, fungi, nematodes and rotifer^10^. However, dietary preferences have been observed to differ between juveniles and adults (juveniles prefer green alga and adults favour rotifers and nematodes^10^).

The ‘Everything is Everywhere’ hypothesis, which was proposed at the beginning of the 20^th^ century^11,12^ suggests that microorganisms and small invertebrates should have a cosmopolitan distribution. Microscopic organisms are often considered cosmopolitan species, as, the presence of specific adaptations allows them to inhabit most environments. These adaptations include a) the possibility of easy passive dispersion (by wind, rivers, sea currents, other animals, etc.), b) the presence of very resistant spore stages (which include cysts, eggs or cryptobiotic individuals) that help to survive extreme conditions, and c) the presence of asexual or parthenogenetic reproduction, allowing for rapid increase in the number of individuals^12,13,14,15,16^. Cosmopolitism was strongly suggested for many tardigrade species in the past, however, the suggestion was later undermined (e.g. ref^17,18,19^). At present, we have strong and compelling evidence of a wide distribution of some tardigrade taxa, which means that we return to the concept of cosmopolitism of at least some species of tardigrades (e.g. ref^8,9,20,21,22^) which can support the hypothesis ‘Everything is Everywhere’ (EiE) for tardigrades. According to Gąsiorek et al^21^, “a species may be termed as cosmopolitan if it was recorded in more than one zoogeographic realm”. There are four tardigrade species known from more than one zoogeographic realm, i.e., *Echiniscus testudo*^23^ (Doyère, 1840), *Milnesium inceptum*^24^ Morek, Suzuki, Schill, Georgiev, Yankova, Marley & Michalczyk, 2019, *Pam. gadabouti*^22^ Kayastha, Stec, Mioduchowska and Kaczmarek. 2023 and *Pam. fairbanksi*. Furthermore, two parthenogenetic species from the genus *Paramacrobiotus*, i.e., *Pam. fairbanksi* and *Pam. gadabouti*, are contenders as they show a wide distribution that supports the hypothesis EiE.

In the present paper we compare different populations of *Pam. fairbanksi* from all known localities of this species in Albania, the Antarctic, Canada, Italy, Mongolia, Poland, Portugal (Madeira) and the USA. We also discuss genetic and morphological differences between them and consider the general distribution of *Pam. fairbanksi*.

## Materials and Methods

### Sample processing

**Figure 1.**
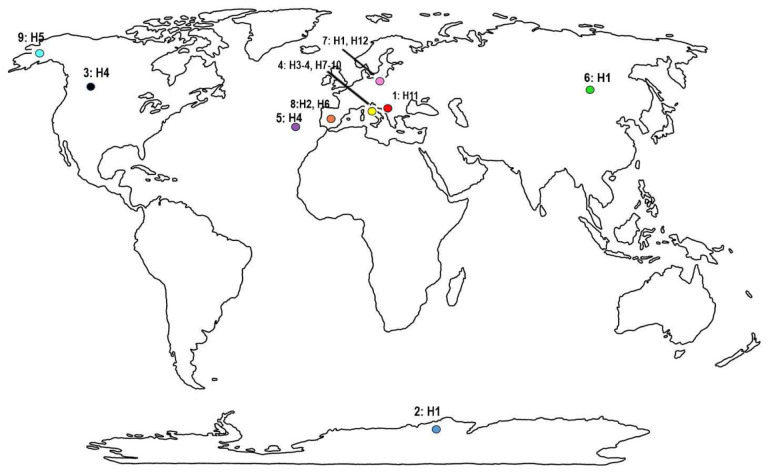
A World map with indicated sample number from Table 1 along with haplotypes of *Paramacrobiotus fairbanksi* Schill, Förster, Dandekar & Wolf 2010^3^ found in different localities (see also Figure 8). The world map is from https://www.wpmap.org/blank-world-map-with-antarctica/blank-world-map-jpg/ and the figure was prepared in Corel Photo-Paint 2021.

Four moss samples from trees and rocks were collected in 2018 (Mongolia) and 2019 (Albania, Canada and Madeira) (for details, see Table 1, Figure 1). The samples were packed in paper envelopes, dried at room temperature and delivered to the laboratory at the Faculty of Biology, Adam Mickiewicz University in Poznań, Poland. Tardigrades were extracted from the samples and studied following the protocol of Stec et al.^25^. The moss samples (Alb, CN8, M85 and MN01) were dried post extractions and were deposited at the Department of Animal Taxonomy and Ecology, Institute of Environmental Biology, Adam Mickiewicz University in Poznań, Uniwersytetu Poznańskiego 6, 61–614 Poznań, Poland.

Additionally, we used morphometric and genetic data of *Pam. fairbanksi* populations from the Antarctic, Italy, Spain, the USA and Poland^9^.

### Culture procedure

Specimens of the populations from Albania, Canada, Madeira and Mongolia were cultured in the Department of Animal Taxonomy and Ecology (Faculty of Biology, Adam Mickiewicz University in Poznań) according to the protocol described by Roszkowska et al.^26^. In summary, tardigrades were cultured in small Petri dishes in spring water mixed with distilled water (1:3) with the rotifers and nematodes added as food *ad libitum*. All cultures were kept in the environmental chamber at a temperature of 18°C and in darkness.

### Microscopy, morphometrics and morphological nomenclature

Specimens were extracted from cultures and prepared for light microscopy analysis. They were mounted on microscope slides in a small drop of Hoyer’s medium and secured with a cover slip^27,28^. Slides were then placed in an incubator and dried for two days at *ca*. 60°C. Dried slides were sealed with transparent nail polish and examined under an Olympus BX41.

All measurements are given in micrometers [μm]. Structures were measured only if their orientation was suitable. Body length was measured from the anterior extremity to the end of the body, excluding the hind legs. Buccal tubes, claws and eggs were measured according to Kaczmarek & Michalczyk^29^. Macroplacoid length sequence is given according to Kaczmarek et al.^30^. The *pt* ratio is the ratio of the length of a given structure to the length of the buccal tube, expressed as a percentage^31^. The *pt* values always provided in italics. Morphometric data were handled using the “Parachela” ver. 1.8 template available from the Tardigrada Register^32^. Tardigrade taxonomy follows Bertolani et al.^33^. Genus abbreviations follow Perry et al.^34^.

### Genotyping

Before genomic DNA extraction, each specimen of *Pam. fairbanksi* was identified *in vivo* using light microscopy (LM). To obtain voucher specimens, DNA extractions were made from individuals using a Chelex^®^ 100 resin (Bio-Rad) extraction method provided by Casquet et al.^35^ with modifications described in Stec et al.^25^. We sequenced three molecular markers, which differ in effective mutation rates: two nuclear fragments (18S rRNA and 28S rRNA) and one mitochondrial fragment (COI). All DNA fragments were amplified according to the protocols described in Kaczmarek et al.^9^, with primers listed in Table 2. Alkaline phosphatase FastAP (1 U/μl, Thermo Scientific) and exonuclease I (20 U/μl, Thermo Scientific) were used to clean the PCR products. Sequencing in both directions was carried out using the BigDyeTM terminator cycle sequencing method and ABI Prism 3130xl Genetic Analyzer (Life Technologies).

### Molecular data analysis

The amplified nuclear and mitochondrial barcode sequences were edited using the BIOEDIT software^39^. Comparison of obtained molecular markers with those deposited in GenBank and homology search were performed using BLAST application (Basic Local Alignment Search Tool^40^). The COI haplotypes were generated using the DnaSP v5.10.01 program^41^ and were translated into amino acid sequences using the EMBOSS-TRANSEQ application^42^ to check for internal stop codons and indels. Then all sequences obtained in our study, and the sequences downloaded from the GenBank database as originating from *Pam. fairbanksi*, were aligned with CLUSTALW using default settings. Alignment sequences were trimmed to 689, 572 and 574 bp for 28S rRNA, 18S rRNA and COI barcodes, respectively. The calculation for the uncorrected pairwise distances (p-distances) was performed for COI sequences using the MEGA X^43^.

All obtained sequences have been deposited in GenBank (for the accession numbers please see Table 3). The slides prepared from exoskeleton/voucher after DNA extraction of *Pam. fairbanksi* were deposited at the Department of Animal Taxonomy and Ecology, Institute of Environmental Biology, Adam Mickiewicz University in Poznań, Uniwersytetu Poznańskiego 6, 61–614 Poznań, Poland.

Reconstruction of genetic relationships among COI haplotypes and genealogical connections was carried out using the Network 4.6.1.3 software (www.fluxuxengineering.com). The median-joining algorithm (MJ)^44^ and substitution rates with the weight of 3 for transitions and 1 for transversions (transition: transversion ratio (ti:tv)) were applied. The star contraction pre-processing was generated to delete all superfluous median vectors and links. Additionally, the maximum parsimony post-processing was calculated. In turn, signatures of population expansion, equilibrium or decline in *Pam. fairbanksi* were inferred from the neutrality tests calculation (Tajima *D*^45^ and Fu *F_S_*^46^, respectively) computed in the DnaSP v5.10.01 program and Arlequin v.3.5. software^47^. Analyses were performed with 1 000 replicates.

### Statistical analysis

We used the Analysis of Variance (ANOVA) test with post hoc comparison of pairs of measurements, applying Bonferroni correction to statistically analyze the differences in morphometrics between different populations of *Pam. fairbanksi*. Measurements of the body and buccal tube length (BL and BTL, respectively) were used as the dependent and the populations as grouping variables. Normal distribution in residuals was checked using the Shapiro test. Other morphometric traits, i.e., stylet support insertion points (SSIP), external width of buccal tube (BTEW) and placoids (M1 – macroplacoid 1, M2 – macroplacoid 2, M3 – macroplacoid 3, Mi – microplacoid, MR – macroplacoid row, PR – placoid row) were also analysed. All the analyses were performed in R 4.1.3^49^. The level of statistical significance was considered at p < 0.05. In the case of post hoc tests, only statistically significant results were presented. Principal Component Analysis (PCA) was performed using the R script from Stec et al.^48^. The analysis was performed for data from eggs and animals. For animals, both absolute values (raw measurements in μm) (BLm, BTLm, SSIPm, BTEWm, M1m, M2m, M3m, Mim, MRm and PRm) and relative *pt* values (BL*pt*, SSIP*pt*, BTEW*pt*, M1*pt*, M2*pt*, M3*pt*, Mi*pt*, MR*pt* and PR*pt*) were used. For eggs, absolute values (raw measurements in μm) were used. All analyses were carried out using the R software program^49^. The “imputePCA” function of the R package “missMDA ver. 1.17” was used to impute missing data in the animal data set using the PCA imputation technique^50^. Cross-validation (function “estim_ncpPCA”) was used to determine the number of components utilized to impute the missing data. The PCA function of the software “FactoMineR ver. 2.3”^51^ was used to perform PCAs on the scaled data. The software “ggplot2 ver. 3.3.2”, “plyr ver. 1.8.6”, and “gridExtra ver. 2.3” were used to depict PCAs^52,53^. The presence of a structure in the PCA data was tested using a randomization approach on the eigenvalues and statistics according to Björklund^54^ and an in-house R script developed by MV in Stec et al.^48^. PERMANOVA was done on the PCs with the R packages “vegan ver. 2.5.6” and “pairwiseAdonis ver. 0.3”^55^, with the species hypothesis generated by phylogenetic techniques as the independent variable. Using the Benjamini-Hochberg correction, the -level for multiple post hoc comparisons was adjusted independently for adult and egg^56^. In total, 106 tardigrade specimens (16 Albanian, 16 Antarctic, 17 Canadian, 15 Madeiran, 14 Mongolian, 15 Polish, 4 Italian and 9 Alaskan) were measured and later used in the analyses for animals. Furthermore, differences in egg morphology between populations were studied and tested using ANOVA. Egg bare diameter (EBD), full diameter (EFD) and processes height (PH) were characters for the populations used as the dependent variable to determine compared groups and Bonferroni corrections. In total, 100 tardigrade eggs (15 Albanian, 16 Antarctic, 15 Canadian, 15 Madeiran, 6 Mongolian, 15 Polish and 18 Alaskan) were measured and used in the analyses. All the analyses were performed in R 4.1.0. The level of statistical significance was considered at p < 0.05. Only statistically significant results were presented for post hoc tests.

### Potential distribution of cosmopolitan Pam. fairbanksi and Pam. gadabouti

A map of the known distribution of *Pam. fairbanksi* populations was assembled in Corel Photo-Paint 2021.

An ecological niche modelling (ENM) approach was used to predict the current potential distribution of *Pam. fairbanksi* and *Pam. gadabouti*. The ENM was performed with the use of the Maxent algorithm, ver. 3.4.4.^48^. MaxEnt performs the model with the fewest possible occurrence data and takes presence-only (PO) data. The model generates models of habitat appropriateness by handling continuous and categorical variables using regularization parameters^57,58^. The raster package in R was used to extract climatic raster values, and for ENM evaluation, version 0.3.1 of ENMevaluate in R was used. The bioclimatic variables available in MERRAclim Dataset 19 were used as environmental variables for Maxent modelling. We used MERRAclim Dataset because it provides a global set of satellite-based bioclimatic variables that includes Antarctica, which is one of the locations for *Pam. fairbanksi*. The 19 global bioclimatic datasets from the 2000s at 5 arcminutes resolution (mean value)^59^ consist of temperature layers (BIO1-BIO11) and humidity layers (BIO12-BIO19). The temperature layers are in degrees Celsius multiplied by 10 and the humidity layers are in kg of water/kg of air multiplied by 100000^59^. The receiver operating characteristic (ROC) plot’s area under curve (AUC) was used to assess the model’s accuracy^57^. AUC describes the relationship between the proportion of correctly-anticipated presences and the proportion of absences of mistakenly-projected species in the model^60^. The AUC gauges the effectiveness of the model with a value between 0 and 1. Furthermore, AUC values > 0.9 indicate excellent accuracy, 0.7 to 0.9 indicate good accuracy, and values below 0.7 indicate low accuracy^57,61,62^. The jackknife test was used to estimate the model’s variable relevance. The localities for *Pam. fairbanksi* are from Table 1 and *Pam. gadabouti* from Kayastha et al.^22^. The coordinate list is provided in SM.01 and the R script for ENM in SM.02.

## 3 Results

### Morphometric comparison of different Pam. fairbanksi populations

**Figure 2.**
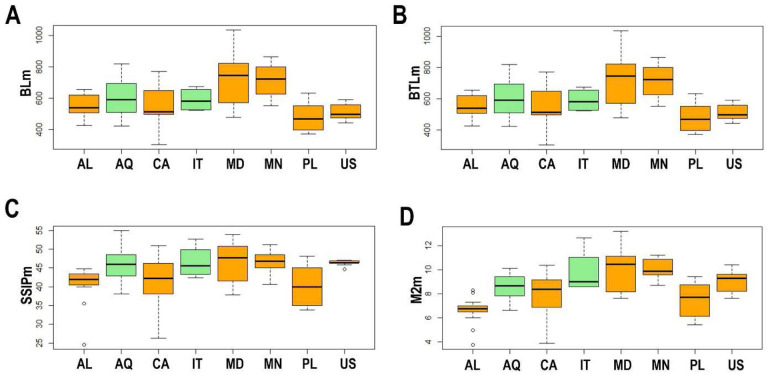
A – Differences in the body length (BLm); B – differences in the buccal tube length (BTLm); C – differences in the stylet support insertion point (SSIPm); D – differences in the Macroplacoid 2 length (M2m). The studied populations of *Paramacrobiotus fairbanksi* Schill, Förster, Dandekar & Wolf 2010 are AL – Albania; AQ – Antarctic; CA – Canada; IT – Italy; MD – Madeira; MN – Mongolia; PL – Poland; US – USA. Minimum, maximum, median, first quartile and third quartile for each population are presented. All measurements are in micrometres [μm]. Orange boxplots represent cultured population and green boxplots represent wild population.

No significant differences were shown by the ANOVA test performed on BL between the studied populations (df = 7; F = 7.832; p = 0.902; N = 106; Table 7, Table 9, Table 11, Table 13; Figure 2A). However, significant differences were found on BTL between different populations (df = 7; F = 5.633; p = 0.010; N = 106; Table 7, Table 9, Table 11, Table 13), where the buccal tube of the specimens from Mongolia was significantly longer than in specimens from the Albanian and Canadian populations (p = 0.002 and p = 0.005 respectively; Figure 2B). The buccal tube of the specimens from Madeira was significantly longer than in specimens from the Polish population (p = 0.003; Figure 2B). Analysis for SSIP length showed significant differences as well (df = 7; F = 4.812; p = 0.016; N = 106; Figure 2C), with the specimens in the Polish population having significantly lower SSIP than in specimens from the Antarctic (p = 0.048), Madeiran (p = 0.038), and Mongolian (p = 0.023) populations. Analysis of M2 length showed significant differences as well (df = 7; F = 8.48; p = 0.020; N = 106; Table 7, Table 9, Table 11, Table 13; Figure 2D).

**Figure 3.**
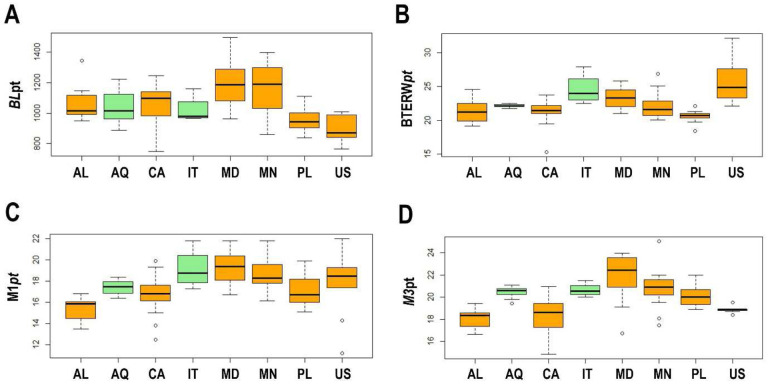
A – Differences in the *pt* body length (BL*pt*); B – differences in the *pt* of external width of buccal tube (BTEW*pt*); C – differences in the *pt* of Macroplacoid 1 (M1*pt*); D – differences in the *pt* of Macroplacoid 3 (M3*pt*). The studied populations of *Paramacrobiotus fairbanksi* Schill, Förster, Dandekar & Wolf 2010 are AL – Albania; AQ – Antarctic; CA – Canada; IT – Italy; MD – Madeira; MN – Mongolia; PL – Poland; US – USA. Minimum, maximum, median, first quartile and third quartile for each population are presented. Orange boxplots represent cultured population and green boxplots represent wild population.

The ANOVA test showed, however, no statistical significance for *pt* of BL between populations (df = 7; F = 8.056; p = 0.678; N = 106; Table 7, Table 9, Table 11, Table 13; Figure 3A). The ANOVA test for *pt* values of the SSIP showed no statistically significant differences between studied populations (df = 7; F = 20.81; p = 0.112; N = 106; Table 7, Table 9, Table 11, Table 13; Figure 3A) whereas the ANOVA test for *pt* values of the BTEW showed differences between studied populations (df = 7; F = 9.87; p = 0.0.001; N = 106; Table 7, Table 9, Table 11, Table 13; Figure 3B). The *pt* values specimens from the Italian population were higher than the Canadian population (p=0.042) and the Polish population (p=0.004), while *pt* values of specimens from the Madeiran population were higher than the Polish population (p=0.003) and *pt* values of specimens from the Alaskan population were higher than the Madeiran population (p=0.018) and the Mongolian population (p=0.0001) (Figure 3B). The ANOVA test for *pt* values of the M1(df = 7; F = 8.38; p = 0.0.007; N = 106; Table 7, Table 9, Table 11, Table 13; Figure 3C) and M3 (df = 7; F = 14.53; p = 0.001; N = 106; Table 7, Table 9, Table 11, Table 13; Figure 3D) showed differences between studied populations.

**Figure 4.**
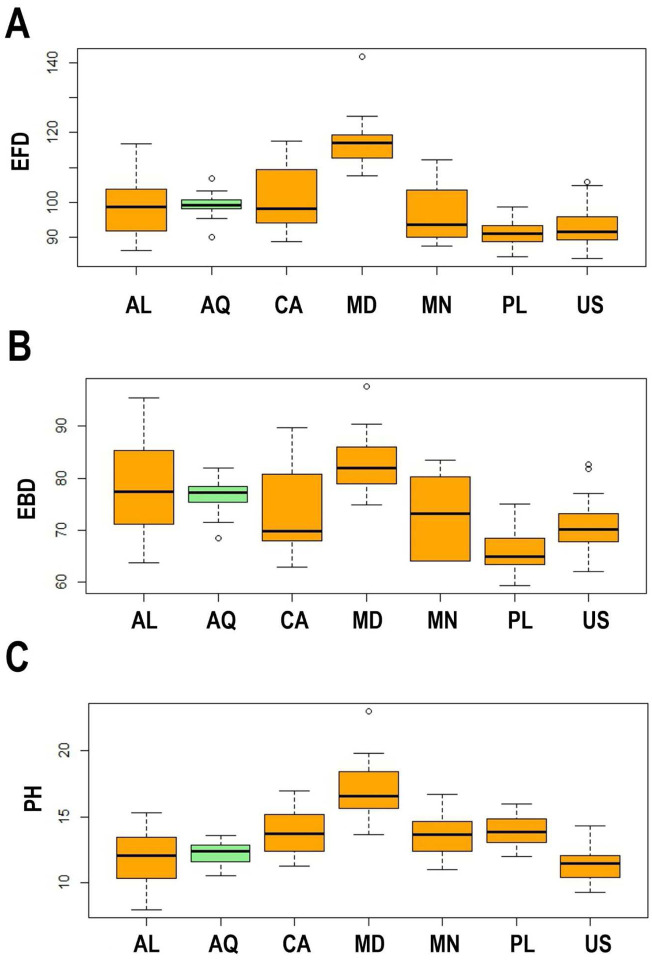
A – Differences in the egg full diameter (EFD); B – differences in the egg bare diameter (EBD); C – differences in the egg processes height (PH). The studied populations of *Paramacrobiotus fairbanksi* Schill, Förster, Dandekar & Wolf 2010 are AL – Albania; AQ – Antarctic; CA – Canada; MD – Madeira; MN – Mongolia; PL – Poland; US – USA. Minimum, maximum, median, first quartile and third quartile for each population are presented. All measurements are in micrometres [μm]. Orange boxplots represent cultured population and green boxplots represent wild population.

The ANOVA performed on EFD measurements of eggs (df = 6; F = 22.92; p = 0.002; N = 100) showed significant differences between all the populations (Table 8, Table 10, Table 12, Table 14). Eggs in the Polish population were significantly smaller than those from Antarctica (p = 0.068) and Canada (p = 0.003), and, eggs from the Alaskan population were clearly smaller than those from Canada (p = 0.008) (Figure 4A). Analysis of EBD values, however, showed no statistically important differences between eggs in different populations (df = 6; F = 9.192; p = 0.249; n = 100; Table 8, Table 10, Table 12, Table 14; Figure 4B). There were also no statistically important differences between the studied populations (Table 8, Table 10, Table 12, Table 14) in the size of egg processes (PH) (df = 6; F = 24.42; p = 0.260; n = 100; Figure 4C).

**Figure 5.**
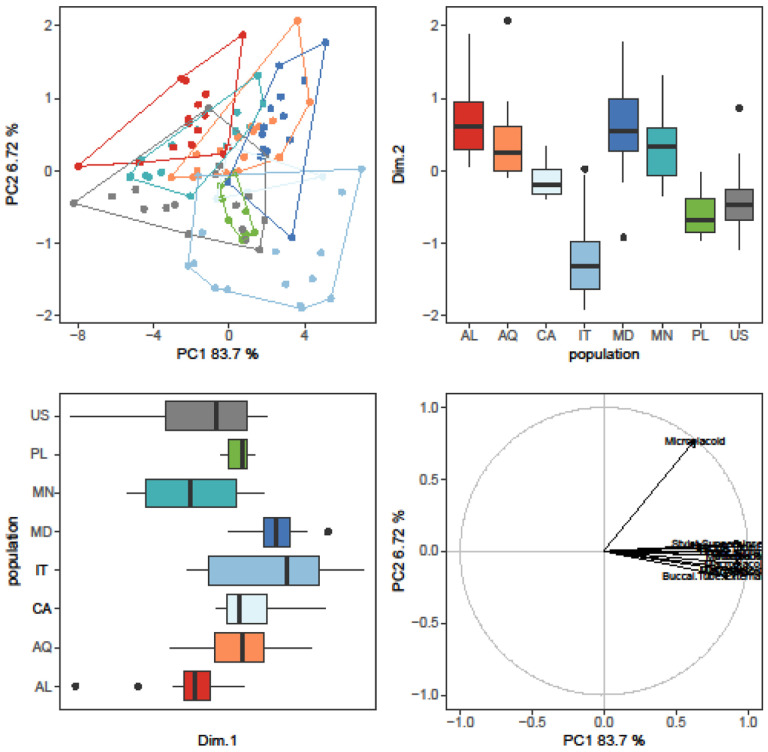
Results of PCA for animal measurements, 1^st^ and 2^nd^ Principal Components. Score scatterplots presented in top-left quadrants; boxplots of single component scores presented in top-right and bottom-left quadrants and loading plot presented in bottom-right.

**Figure 6.**
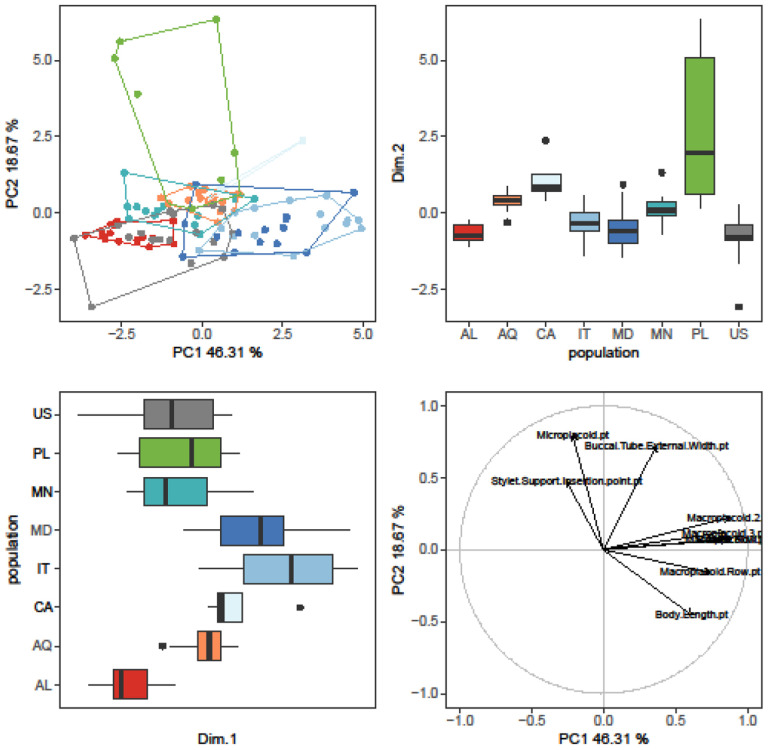
Results of PCA for animal *pt* indices, 1^st^ and 2^nd^ Principal Components. Score scatterplots presented in top-left quadrants; boxplots of single component scores presented in top-right and bottom-left quadrants and loading plot presented in bottom-right.

**Figure 7.**
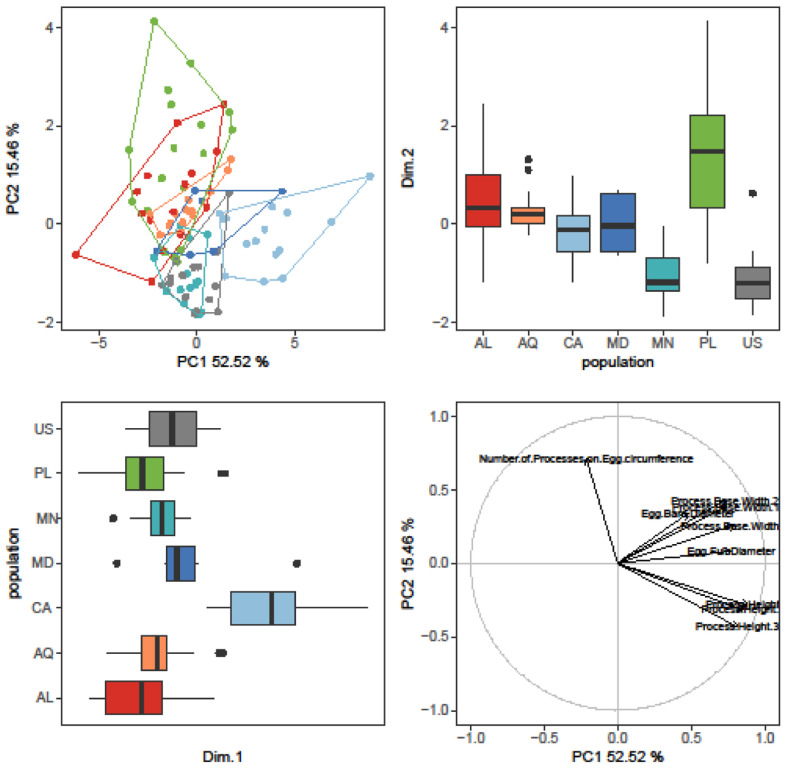
Results of PCA for egg measurements, 1^st^ and 2^nd^ Principal Components. Score scatterplots presented in top-left quadrants; boxplots of single component scores presented in top-right and bottom-left quadrants and loading plot presented in bottom-right.

The randomization test in PCA demonstrated that only the first two PCs explained greater variation than was anticipated by the null model (no data structure) for both animal and egg datasets (SM.11). As a result, only the initial two PCs were maintained and used for additional investigation and interpretation. Furthermore, the ψ and ϕ statistics of the PCA were significantly distinct from what they anticipated under the null assumption (animals: ψ=60.72 p<0.001, ϕ=0.82 p<0.001; animals *pt*: ψ=13.14 p<0.001, ϕ=0.43 p<0.001; eggs: ψ=17.62 p<0.001, ϕ=0.50 p<0.001). The first two components of the PCA of animals’ absolute measured value (Figure 5) explained 90% of the overall variation (83.7% for PC1 and 6.7% for PC2) and for animals’ *pt* indices (Figure 6) explained 65% of the overall variation (46.3% for PC1 and 18.7% for PC2). PCA of egg measurements (Figure 7) described 68% of the total variance with the first two components (52.5% for PC1 and 15.5% for PC2). PERMANOVA revealed that species identity has a substantial overall effect on PCs (p<0.001, Table 4, Table 5, Table 6). Raw morphometric data for all the populations in the present study are given in the Supplementary Materials (SM.03-SM.06). R script for single characters as well as measurement files for both adults and eggs, are provided in the Supplementary Materials (SM.07-SM.09). All the test results from R are provided in Supplementary Materials (SM.10). Results of PCA randomization tests in the Supplementary Materials (SM.11).

### Genetic comparisons and phylogeographical analyses of different populations of the Pam. fairbanksi

**Figure 8.**
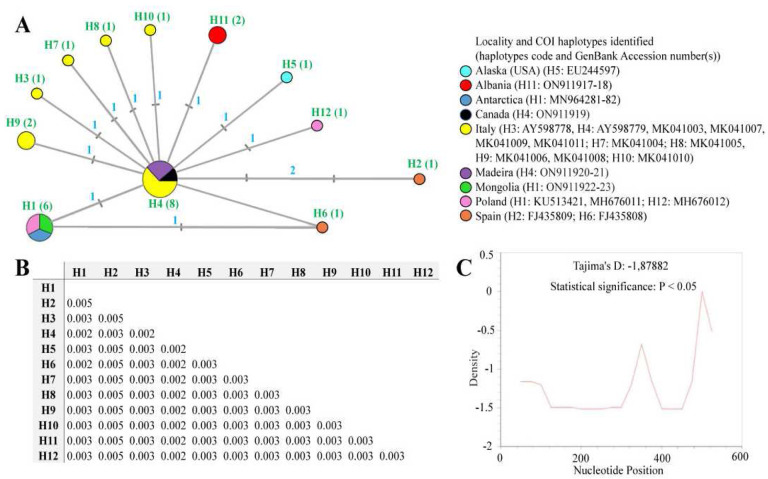
A – Median-joining network based on the COI sequences: haplotypes marked as H1-H12 (the number of sequences is given in parentheses), the size of the circles is proportional to the number of sequences, the mutational steps values are indicated along the lines; B – p-distance value based on the COI barcode sequences; C – Tajima’s D neutrality test.

The COI sequences of *Pam. fairbanksi* from Albania, Canada, Madeira and Mongolia were 623–689 bp-long, and represented three haplotypes: haplotype H11 was observed in the population from Albania, haplotype H1 was identified in *Pam. fairbanksi* from Mongolia, and haplotype H4 was found in populations from Canada and Madeira (for details see Table 3 and Figure 8A, B). No stop codons, insertions or deletions were observed. The translation was successfully carried out with the −2^nd^ reading frame and the invertebrate mitochondrial codon table. The p-distances between COI haplotypes of all sequenced *Pam. Fairbanksi* populations deposited in GenBank, i.e., from Antarctica, Italy, Spain, the USA and Poland ranged from 0.002% to 0.005% (an average distance of 0.003%) (Figure 8B). In total, twelve haplotypes (H1-H12) of COI gene fragments were identified after comparing all available COI sequences of *Pam. fairbanksi*. Overall, the median joining COI haplotype network showed a star-like radiation. Interestingly, the most frequent haplotype H4 was present in populations from Italy, Madeira and Canada. This central haplotype H4 was surrounded by ten haplotypes (H1, H3, H5-H12) that differed from it by one mutational step. One haplotype (haplotype H2 from Spain) differed from central haplotype H4 by two mutational steps. In several geographical regions, i.e., the USA, Albania, Italy, Poland and Spain there were regional endemic haplotypes. Surprisingly, the second haplotype that occurred in different localities was haplotype H1 and this haplotype was common for three populations, from Mongolia, Poland and Antarctica.

In turn, the 18S rRNA sequences of *Pam. fairbanksi* from Albania, Canada, Madeira and Mongolia were 917–1547 bp-long (Table 3) and no nucleotide substitution was found (although a single “N” was identified, i.e. software was unable to identify this base). Compared with the data available in GenBank sequences of *Pam. fairbanksi* (sequences were alignment and trimmed to 572 bp), they showed only one nucleotide substitution. A comparison was performed with the sequences from the following geographical localities: Antarctica (GenBank: MN960302^9^), Poland (GenBank: MH664941-42^63^), USA (GenBank: EU038078^64^) and Italy (GenBank: MK041027-29^8^). The 28S rRNA molecular marker was very conservative, and was 694–805 bp-long. No nucleotide substitution was found for all obtained sequences even after comparing (and trimmed to 689 bp) with GenBank sequences from Antarctica (GenBank: MN960306 – MN960307^9^) and Poland (GenBank: MH664950^63^). Nevertheless, one unidentified base was found in the sequence originating from the Polish population.

Demographic expansion was preliminarily tested based on the value of neutrality tests that confirmed a neutral model of observed polymorphism. Negative significant values for Tajima’s D were found, indicating a high number of low-frequency polymorphisms in the COI sequences dataset and potential population size expansion (Figure 8C). In turn, values of Fu’s FS test statistic for COI data were negative, but non-significant: −0.25702, P = 1.16679 (graphical results not shown).

### Predictions of the distribution of the two parthenogenetic Paramacrobiotus species

**Figure 9.**
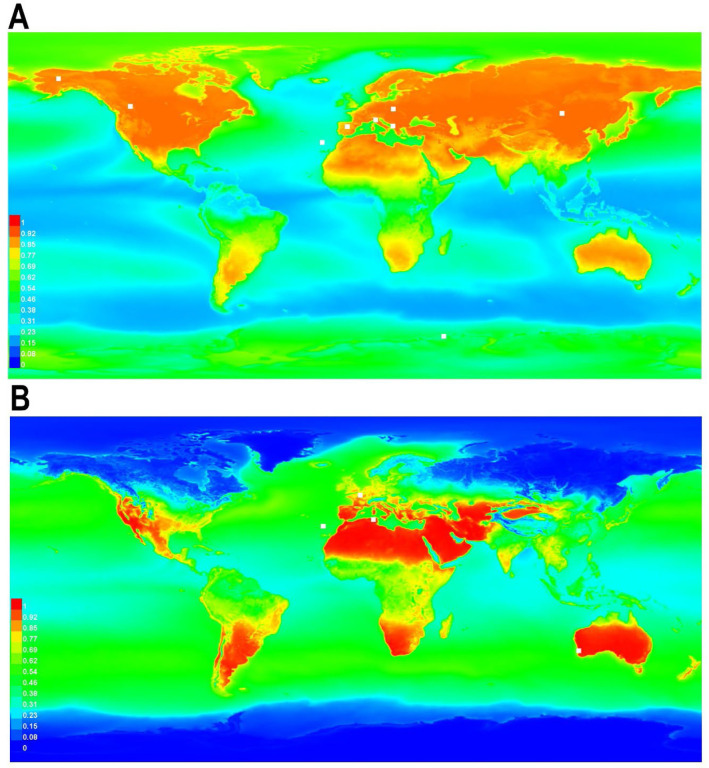
Ecological biogeography of two parthenogenetic *Paramacrobiotus* species with wide distributions – geographic ranges predicted by ecological niche modelling for: (A) *Paramacrobiotus fairbanksi* Schill, Förster, Dandekar & Wolf 2010^9^, (B) *Paramacrobiotus gadabouti* Kayastha, Stec, Mioduchowska and Kaczmarek 2023^21^. Suitability determines whether a given area is characterised by favourable conditions for one of the species (maximal suitability = 1) or by allegedly inhospitable conditions (minimal suitability = 0). Generated using Maxent, ver. 3.4.4 [https://biodiversityinformatics.amnh.org/open_source/maxent/]. Warmer colours show areas with better predicted conditions. White dots show the presence locations used for training.. The maps were generated using MaxEnt software ver. 3.2.0: https://biodiversityinformatics.amnh.org/open_source/maxent/ and assembled in Corel Photo-Paint 2021.

Ecological niche modelling of potential distribution based on available location data was performed for two parthenogenetic species with verified records from various realms, i.e., *Pam. fairbanksi* and *Pam gadabouti*. The study is limited to bioclimatic variables. The stimulated model predicted good accuracy for the overall model with an AUC for *Pam. fairbanksi* of 0.826 and excellent accuracy for the overall model with an AUC for *Pam gadabouti* of 0.924. The suitability for *Pam. fairbanksi* seems moderate (green areas on the map in Figure 9A) to good (yellow areas on the map in Figure 9A) with the most suitable habitats in the northern hemisphere. *Pam. gadabouti* shows maximal suitability around areas with a Mediterranean climate, although it also has wide distribution (Figure 9B).

## 4. Discussion

### Morphometric comparison of different populations of the Pam. fairbanksi

Based on morphometric analyses, there is clearly a variation in measurements of morphological features between populations of *Pam. fairbanksi* from different regions of the world. However, the identification of this species is still possible with the morphometric characters alone because of the overlap in measurements of all measured structures. Therefore, it is valid to suggest the correct classification of all the specimens collected from different regions based on their morphology only. Even though the egg processes of Polish and Albanian populations are similar, the EBD of the Polish population are the smallest and those from the Albania are largest. The EFD as well as egg processes of the Madeiran population are largest while those of the Polish population are the smallest. Additionally, body length values of the Polish and USA populations of *Pam. fairbanksi* are smaller compared to the other populations studied.

Kaczmarek et al.^9^ suggested that the differences in measurements between different populations of this species are caused by conditions, i.e., specimens from cultures and specimens from wild populations. However, in the present study all measurements were based on specimens from cultured populations, i.e., Albanian, Canadian, Madeiran and Mongolian. Thus, we can suggest that the phenomenon described by Kaczmarek et al.^9^ (that dwarfing is caused by suboptimal conditions, high culture densities and inbreeding and that it might be due to the result of ongoing speciation) is unlikely to be true. Similarly, the suggestions that harsh conditions in Antarctica may favor laying larger eggs while in cultures the eggs are smaller because of the lack of such selective pressure^9^ seems untrue as the egg size of specimens from Antarctica overlaps with egg sizes of specimens from Albania, Canada and Mongolia, which were sampled from cultured populations in the present study.

### Genetic comparison of different populations of the Pam. fairbanksi

Cytochrome oxidase subunit I gene (COI) sequences is one of the most reliable barcodes to investigate genetic variation with phenotypic plasticity since COI is a genetic marker with a high genetic variation compared to multiple other DNA barcodes^66^. Various studies combining COI variation and phenotypic plasticity were conducted throughout different invertebrates’ phyla, including tardigrades^9,21,66,67,68,69^, proving the marker’s accuracy in this group of organisms. The result showed high genetic homogeneity between organisms with wide geographical distribution together with clearly visible morphological differences known as phenotypic plasticity^67^.

Furthermore, several studies uncovered data incongruence between mitochondrial and nuclear markers, e.g., for earthworms^70^ or corals^71^, suggesting that occasionally COI may fail as a barcode marker due to hybridization events. Many studies have already shown that *Wolbachia* (presence shown by Mioduchowska et al^72^ in *Pam. fairbanksi*) can increase the speciation rate and can affect COI haplotypes^73^. However, the nuclear markers tested for *Pam. fairbanksi* have been consistent for the studied populations. No molecular markers that correspond with morphological features in tardigrades have been suggested so far. Future studies with higher-resolution markers designed for intrapopulation variation should be performed to determine if any pattern of genetic diversity concordant with morphological variation can be observed.

Based on morphometric and genetic analyses, it is possible that the subtle morphological variation observed in geographically remote populations of *Pam*. *fairbanksi* can be explained by phenotypic plasticity. This explanation is supported by the fact that populations from Poland and Antarctica share the same haplotype, H1, but vary in EFD measurements of eggs, which is the most variable trait in the analysis. Several distant geographic populations share the same COI haplotype, H1 or H4, yet it does not make them morphologically consistent within the haplotypes. These populations have, as shown in the study, a low evolutionary rate, and the inter-population variation develops under variable conditions experienced in different locations. The exact causes and mechanisms of the phenotypic plasticity in the morphology of adults and eggs of *Pam*. *fairbanksi* remains unknown, although, it has unsurprisingly been shown, that some physical traits differ in chosen cultured tardigrades depending on the temperature and food abundance^7^. If the morphological variation in *Pam. fairbanksi* is an effect of phenotypic plasticity, it is unclear which factors could cause various morphotype expressions. The specimens from Mongolia, Albania, Canada and Madeira that were measured in our study come from populations cultured in similar laboratory conditions but were started with different counts of founders of various ages, kept in variable densities and with different numbers of generations that had passed culture, so no answer can be proposed at this moment.

Phenotypic plasticity, in morphology and other aspects of phenotype, such as life history traits, is seen as an advantage for thriving in heterogeneous environments (e.g. ref^74^), which tardigrades’ habitats clearly are. Furthermore, phenotypic plasticity has been widely observed in other invertebrates like corals (e.g. *Pseudopterogorgia bipinnata* Verrill 1864^75^), scallops (e.g. *Pecten maximus* Linnaeus, 1758^76^), marine invertebrates, gastropods (e.g. *Littorina littorea* Linnaeus, 1758^62^), rotifers (*Keratella tropica* (Apstein, 1907)^77^) and many more. No concordant genetic variation was observed, but a large and discrete differentiation of morphotypes was present and was always associated with external environmental factors such as temperature, predation risk and food availability^78.79,80,82,82^.

### Parthenogenesis and wide distribution

The phenomenon where parthenogenetic (asexual) lineages occupy a wider geographical range, but sexual populations are restricted to a limited area, is termed ‘geographical parthenogenesis’^83^. Guidetti et al.^8^ concluded that the difference in the dispersal potential of tardigrades is associated with the two types of reproduction, i.e., parthenogenetic species show a very wide distribution, inhabiting more continents, while the amphimictic species show a very limited or punctiform distribution. A similar pattern was shown for arthropods where parthenogenesis has been linked with higher dispersal abilities^84^ (for example, the freshwater ostracod *Eucypris virens* (Jurine, 1820)^85^ and the scorpion species *Liocheles australasiae* (Fabricus 1775) are parthenogenetic for multiple generations in captivity^86,87^ and are widely distributed^88,89^. Similar cases are found in many animals and plants (ref^90,91,92,93^)). However, Baker et al.^84^ also suggested that parthenogenesis indicates morphological variation as a result of epigenetic mechanisms. Furthermore, Mioduchowska et al.^72^ provided molecular evidence of the presence of the bacterial endosymbiont *Wolbachia* based on next generation sequencing in tardigrades. *Wolbachia* have an effect on the evolution as well as the ecology of their hosts, and have been found to cause effects including cytoplasmic incompatibility, feminization, male killing, and induced parthenogenesis^94^ It has been noted that at the intraspecific level, even individuals from the same population can undergo morphological changes in their characters to diversify within niches available to the species^95^. Similarly, Kihm et al.^88^ proposed epigenetic factors as a main cause for variability in tardigrade *Dactylobiotus ovimutans* egg morphology, although the population was cultured under controlled laboratory conditions. Despite being rare, it is known that intraspecific variation is caused by external environmental conditions, epigenetics and seasonality^96^. Therefore, it is also likely that the morphological differences that we observed in the present study might be due to epigenetic factors, as the studied populations were cultured under controlled laboratory conditions.

### “Two faces” of cosmopolitism in the Paramacrobiotus

Ecological niche modelling is an important and useful tool that has been used to address issues in many fields of basic and applied ecology^97^. It effectively predicts habitat suitability for rare and poorly studied taxa^98,99^. *Pam. fairbanksi* presence is linked to the presence of suitable microhabitats, like moss patches, and their life strategy can make them less likely to be affected by general climatic conditions. However, bioclimatic variables used in the study may be a good predictor of the possibility of the occurrence of suitable microhabitats. We investigated the possible distribution of *Pam. fairbanksi* and compared it with other widely distributed species of the genus *Paramacrobiotus*, i.e., *Pam. gadabouti*. *Paramacrobiotus fairbanksi* already reported from various continents exhibit a cosmopolitan distribution covering different types of environments, whereas *Pam. gadabouti*, although also potentially cosmopolitan, has a clear affinity to areas with a Mediterranean climate. Its distribution is poorly known due to lack of sampling in many habitats. Such differences clearly show us that even when we consider some of the species to be cosmopolitan, specific patterns of distribution can be completely different. However, we must also stress that the number of known localities for both species is relatively low and, in the future, when the number of records of these species will be higher, a distribution pattern may look different.

## Conclusions

*Paramacrobiotus fairbanksi* described originally from Alaska, USA, is now known from almost all zoogeographic realms. The identification of this species is possible based on morphometric characters alone because of the overlap in measurements of all measured structures. Moreover, the analysis shows low genetic variability among *Pam. fairbanksi* populations from various geographical locations, which may in general suggest that interspecies genetic variability in tardigrades is very low too or could be’ the effects of *Wolbachia* infection. The species fits the ‘Everything is Everywhere’ hypothesis and is an example of a parthenogenetic species with wide distribution. Despite very low genetic variation, some indiscrete morphological variations were observed. Since all the studied populations were cultured and bred in the same laboratory conditions, such variation may have been caused by epigenetic effects, and were not the result of different temperatures, food sources and seasonality.

## Figures and Tables

**Table 1.** Studied populations of *Paramacrobiotus fairbanksi* Schill, Förster, Dandekar & Wolf, 2010^3^ (see also Figure 1).

Sample No	Coordinates	Locality and sample description	Remarks
1	41°19’36”N, 19°49’08”E; 112 m asl	Albania, Tirana County, Tirana, near Bunk’Art 2; moss on tree	Present study
2	*ca*. 67°39'S, 46°09'E; 0 m asl	Antarctic, near Vechernia Mt Base; moss (*Ceratodon purpureus*)	Kaczmarek et al.^9^
3	51°24’21”N, 116°14’27”W; 1900 m asl	Canada, Alberta, Banff National Park, near east end of the Louise Lake; moss on stone	Present study
4	*ca*. 44°26’N, 10°51’E; 510 m asl	Ricco, Modena Province, Italy; beech leaf litter	Kaczmarek et al.^9^
5	32°44’37.3”N, 16°54’14.4”W; 710 m asl	Portugal, Madeira, Ribera de Brava; moss on rock	Present study
6	47°49’57.0”N, 107°31’26.8”E; 1432 m asl	Mongolia, Tov Province; moss on rocky hill	Roszkowska et al.^26^
7	50°03’44”N, 19°57’26”E; 205 m asl	Poland, Lesser Poland Province, Kraków, Jagiellonian University Botanical Garden, Kopernika 27 street; moss on tree	Kaczmarek et al.^9^
8	40°52′42″N, 03°50′45″W; asl	Spain, Madrid; litter, oaks	Guil and Giribet^6^
9	*ca*. 64°50’N, 147°43’E; 135 m asl	USA, Alaska, Fairbanks; moss	Kaczmarek et al.^9^

**Table 2.** Primers with their original references used for sequencing of three molecular markers of *Paramacrobiotus fairbanksi*.

DNA molecular marker	Primer name and direction	Primer sequence (5’-3’)	Source
COI	LC01490 (forward) HCO2198 (reverse)	GGTCAACAAATCATAAAGATATTGG TAAACTTCAGGGTGACCAAAAAATCA	36
18S rRNA	SSU01_F (forward) SSU82_R (reverse)	AACCTGGTTGATCCTGCCAGT TGATCCTTCTGCAGGTTCACCTAC	37
28S rRNA	28SF0001 (forward) 28SR0990 (reverse)	ACCCvCynAATTTAAGCATAT CCTTGGTCCGTGTTTCAAGAC	38

**Table 3.** GenBank accession numbers of sequences obtained in the present study along with the slide numbers of voucher specimens.

Populations of *Paramacrobiotus fairbanksi*	GenBank accession number; bp long DNA molecules			Voucher numbers
	COI mtDNA	18S rRNA	28S rRNA	
Albania	ON911917-18;	ON872386;	ON872380-81;	Alb2/S,
	623–678	1480	805	Alb3/S,
				Alb4/S
Canada	ON911919;	ON872387;	ON872382;	CN8.2/S
	625	1480	793	
Madeira	0N911920-21;	ON872388;	ON872383;	M85.11/S,
	678–679	1547	744	M85.12/S
Mongolia	0N911922-23;	ON872389;	ON872384-85;	MN0101/S,
	687–689	917	694–711	MN0103/S

**Table 4.** Results of PERMANOVA and post hoc pairwise PERMANOVA comparisons for the first two principal components (PC1 and PC2) of animal measured values; significant post hoc p-values adjusted with the Benjamini-Hochberg correction.

*Post hoc* comparisons	df	SS	F	R^2^	P
Poland vs Italy	1	33.92	4.98	0.23	0.068
Poland vs USA	1	36.99	8.09	0.27	0.019
Poland vs Antarctica	1	49.69	8.77	0.23	0.017
Poland vs Albania	1	0.98	0.17	0.01	0.704
Poland vs Canada	1	5.39	0.65	0.02	0.485
Poland vs Madeira	1	144.31	16.56	0.37	0.003
Poland vs Mongolia	1	143.25	31.67	0.54	0.001
Italy vs USA	1	2.71	1.29	0.11	0.344
Italy vs Antarctica	1	2.50	0.52	0.03	0.502
Italy vs Albania	1	37.72	7.93	0.31	0.019
Italy vs Canada	1	26.61	2.96	0.13	0.145
Italy vs Madeira	1	6.09	0.62	0.04	0.496
Italy vs Mongolia	1	5.94	2.13	0.12	0.207
USA vs Antarctica	1	6.00	1.92	0.08	0.214
USA vs Albania	1	44.90	14.65	0.39	0.002
USA vs Canada	1	23.65	3.64	0.13	0.098
USA vs Madeira	1	18.70	2.72	0.11	0.150
USA vs Mongolia	1	30.82	21.91	0.51	0.001
Antarctica vs Albania	1	55.76	12.46	0.29	0.003
Antarctica vs Canada	1	42.92	6.06	0.16	0.033
Antarctica vs Madeira	1	39.28	5.29	0.15	0.041
Antarctica vs Mongolia	1	27.43	8.23	0.23	0.017
Albania vs Canada	1	11.33	1.61	0.05	0.256
Albania vs Madeira	1	164.10	22.24	0.43	0.001
Albania vs Mongolia	1	153.60	46.73	0.63	0.001
Canada vs Madeira	1	115.28	11.55	0.28	0.005
Canada vs Mongolia	1	133.00	21.73	0.43	0.001
Madeira vs Mongolia	1	23.85	3.72	0.12	0.085

**Table 5.** Results of PERMANOVA and post hoc pairwise PERMANOVA comparisons for the first two principal components (PC1 and PC2) of animal *pt* values; significant post hoc p-values adjusted with the Benjamini-Hochberg correction.

*Post hoc* comparisons	df	SS	F	R^2^	P
Poland vs Italy	1	14.60	7.12	0.30	0.0147
Poland vs USA	1	39.29	9.12	0.29	0.0016
Poland vs Antarctica	1	6.80	5.60	0.16	0.0218
Poland vs Albania	1	23.07	17.70	0.38	0.0002
Poland vs Canada	1	7.41	3.11	0.09	0.0620
Poland vs Madeira	1	96.12	37.76	0.57	0.0002
Poland vs Mongolia	1	45.99	19.38	0.42	0.0002
Italy vs USA	1	15.96	2.33	0.18	0.1088
Italy vs Antarctica	1	5.00	5.75	0.24	0.0214
Italy vs Albania	1	48.07	47.57	0.73	0.0003
Italy vs Canada	1	26.96	9.90	0.34	0.0025
Italy vs Madeira	1	15.25	5.02	0.23	0.0264
Italy vs Mongolia	1	9.39	3.38	0.17	0.0591
USA vs Antarctica	1	36.20	11.01	0.32	0.0013
USA vs Albania	1	85.73	25.23	0.52	0.0002
USA vs Canada	1	76.01	16.33	0.40	0.0002
USA vs Madeira	1	118.93	23.44	0.52	0.0002
USA vs Mongolia	1	88.02	17.70	0.46	0.0002
Antarctica vs Albania	1	55.98	90.28	0.75	0.0002
Antarctica vs Canada	1	22.28	13.23	0.30	0.0002
Antarctica vs Madeira	1	58.07	32.33	0.53	0.0002
Antarctica vs Mongolia	1	23.56	14.70	0.34	0.0003
Albania vs Canada	1	13.26	7.51	0.20	0.0080
Albania vs Madeira	1	199.01	105.68	0.78	0.0002
Albania vs Mongolia	1	116.03	68.54	0.71	0.0002
Canada vs Madeira	1	116.25	39.54	0.57	0.0002
Canada vs Mongolia	1	55.41	19.84	0.41	0.0002
Madeira vs Mongolia	1	9.33	3.12	0.10	0.0783

**Table 6.** Results of PERMANOVA and post hoc pairwise PERMANOVA comparisons for the first two principal components (PC1 and PC2) of animal *pt* values; significant post hoc p-values adjusted with the Benjamini-Hochberg correction.

*Post hoc* comparisons	df	SS	F	R^2^	P
Poland vs USA	1	47.08	17.87	0.37	0.0002
Poland vs Antarctica	1	13.96	12.27	0.30	0.0002
Poland vs Albania	1	25.55	9.43	0.25	0.0002
Poland vs Canada	1	1.19	1.04	0.04	0.3524
Poland vs Madeira	1	134.43	47.34	0.63	0.0002
Poland vs Mongolia	1	9.14	4.73	0.20	0.0254
USA vs Antarctica	1	10.77	3.79	0.11	0.0372
USA vs Albania	1	6.23	1.44	0.04	0.2496
USA vs Canada	1	54.20	18.64	0.38	0.0002
USA vs Madeira	1	198.41	44.72	0.59	0.0002
USA vs Mongolia	1	17.89	4.16	0.16	0.0294
Antarctica vs Albania	1	7.47	2.54	0.08	0.1235
Antarctica vs Canada	1	16.51	11.55	0.28	0.0004
Antarctica vs Madeira	1	135.88	44.34	0.60	0.0002
Antarctica vs Mongolia	1	4.74	2.06	0.09	0.1731
Albania vs Canada	1	33.87	11.25	0.29	0.0004
Albania vs Madeira	1	200.18	42.54	0.60	0.0002
Albania vs Mongolia	1	17.57	3.75	0.16	0.0697
Canada vs Madeira	1	111.85	35.60	0.56	0.0002
Canada vs Mongolia	1	7.01	2.95	0.13	0.0893
Madeira vs Mongolia	1	42.85	8.80	0.32	0.0105

**Table 7.** Measurements [in μm] and pt values of selected morphological structures of individuals of *Paramacrobiotus fairbanksi* Schill, Förster, Dandekar & Wolf 2010 Albanian population mounted in Hoyer’s medium (N – number of specimens/structures measured; RANGE refers to the smallest and the largest structure among all measured specimens; SD – standard deviation, *pt* – ratio of the length of a given structure to the length of the buccal tube expressed as a percentage).

CHARACTER	N	RANGE		MEAN		SD	
		μm	*pt*	μm	*pt*	μm	*pt*
Body length	16	426 – 654	–	549		73	
Buccopharyngeal tube							
Buccal tube length	16	31.7 – 57.8	–	52.1	–	6.4	–
Stylet support insertion point	15	24.6 – 44.8	*76.1 – 80.8*	40.7	*78.7*	5.0	*1.4*
Buccal tube external width	15	7.5 – 14.2	*19.2 – 24.6*	11.1	*21.4*	1.8	*1.8*
Buccal tube internal width	15	5.5 – 10.9	*16.1 – 19.8*	9.0	*17.2*	1.3	*1.0*
Ventral lamina length	14	18.2 – 34.1	*57.4 – 61.5*	30.3	*58.7*	4.1	*1.3*
Placoid lengths							
Macroplacoid 1	15	6.1 – 9.6	*13.5 – 16.8*	8.2	*15.3*	1.0	*1.1*
Macroplacoid 2	16	3.8 – 8.3	*11.0 – 14.5*	6.6	*12.7*	1.1	*0.9*
Macroplacoid 3	16	5.8 – 11.1	*16.6 – 19.4*	9.4	*18.0*	1.3	*0.9*
Microplacoid	16	3.5 – 6.3	*8.3 – 10.9*	4.8	*9.2*	0.6	*0.9*
Macroplacoid row	15	16.8 – 32.0	*50.6 – 56.1*	27.2	*52.5*	3.6	*1.5*
Placoid row	15	21.2 – 40.4	*66.0 – 71.0*	35.3	*68.2*	4.8	*1.4*
Claw 1 heights							
External primary branch	16	10.1 – 16.0	*23.4 – 31.9*	13.9	*26.9*	1.6	*2.2*
External secondary branch	16	7.2 – 13.2	*18.7 – 24.3*	10.7	*20.6*	1.4	*1.5*
Internal primary branch	16	9.5 – 17.3	*24.1 – 30.0*	13.4	*25.8*	1.7	*2.0*
Internal secondary branch	16	6.6 – 13.7	*18.5 – 23.6*	10.6	*20.4*	1.6	*1.4*
Claw 2 heights							
External primary branch	16	10.3 – 18.3	*24.6 – 32.6*	14.8	*28.5*	1.8	*2.1*
External secondary branch	16	8.2 – 13.3	*19.8 – 25.8*	11.5	*22.2*	1.4	*1.4*
Internal primary branch	16	9.8 – 16.6	*23.9 – 31.1*	13.5	*26.1*	1.6	*1.8*
Internal secondary branch	15	8.2 – 13.8	*16.7 – 25.9*	10.7	*20.7*	1.6	*2.3*
Claw 3 heights							
External primary branch	16	11.5 – 19.0	*27.9 – 36.5*	15.4	*29.7*	1.6	*2.2*
External secondary branch	16	8.9 – 14.3	*20.1 – 28.1*	11.8	*22.7*	1.4	*2.3*
Internal primary branch	16	9.7 – 15.9	*23.7 – 30.7*	13.5	*26.0*	1.5	*1.7*
Internal secondary branch	15	7.7 – 13.1	*18.2 – 24.2*	10.9	*21.0*	1.3	*1.6*
Claw 4 heights							
Anterior primary branch	16	11.5 – 20.0	*26.3 – 36.3*	15.8	*30.4*	1.9	*2.5*
Anterior secondary branch	16	8.3 – 14.4	*20.0 – 26.1*	11.9	*22.9*	1.7	*1.8*
Posterior primary branch	16	11.9 – 20.5	*28.4 – 37.6*	16.0	*30.9*	1.9	*2.5*
Posterior secondary branch	16	7.4 – 14.2	*20.7 – 25.7*	12.2	*23.4*	1.6	*1.3*

**Table 8.** Measurements [in μm] of selected morphological structures of eggs of *Paramacrobiotus fairbanksi* Schill, Förster, Dandekar & Wolf 2010 Albanian population mounted in Hoyer’s medium (N – number of specimens/structures measured, RANGE refers to the smallest and the largest structure among all measured eggs; SD – standard deviation).

CHARACTER	N	RANGE	MEAN	SD
Egg bare diameter	15	63.8 – 95.4	77.9	9.3
Egg full diameter	15	86.2 – 116.9	98.5	8.3
Process height	45	7.6 – 17.3	12.0	2.0
Process base width	45	9.7 – 20.3	15.5	2.2
Process base/height ratio	45	100% – 177%	130%	17%
Inter-process distance	42	1.3 – 7.8	3.6	1.6
Number of processes on the egg circumference	15	13 – 16	14.1	1.1

**Table 9.** Measurements [in μm] and pt values of selected morphological structures of individuals of *Paramacrobiotus fairbanksi* Schill, Förster, Dandekar & Wolf 2010 Canadian population mounted in Hoyer’s medium (N – number of specimens/structures measured; RANGE refers to the smallest and the largest structure among all measured specimens; SD – standard deviation, *pt* – ratio of the length of a given structure to the length of the buccal tube expressed as a percentage).

CHARACTER	N	RANGE		MEAN		SD	
		μm	*pt*	μm	*pt*	μm	*pt*
Body length	17	303 – 772	–	564		133	
Buccopharyngeal tube							
Buccal tube length	17	33.8 – 63.7	–	52.7	–	8.6	–
Stylet support insertion point	17	26.3 – 51.0	*75.3 – 80.0*	41.2	*78.3*	6.8	*1.2*
Buccal tube external width	17	6.2 – 13.7	*15.4 – 23.8*	11.3	*21.3*	2.3	*1.9*
Buccal tube internal width	17	4.5 – 11.4	*11.0 – 18.1*	8.2	*15.4*	1.9	*1.8*
Ventral lamina length	15	24.5 – 38.3	*56.4 – 64.7*	32.6	*60.4*	4.9	*2.6*
Placoid lengths							
Macroplacoid 1	17	4.7 – 11.7	*12.5 – 19.9*	8.9	*16.7*	2.1	*1.8*
Macroplacoid 2	17	3.9 – 10.4	*11.2 – 16.8*	7.8	*14.6*	1.9	*1.6*
Macroplacoid 3	17	5.0 – 12.1	*14.9 – 20.9*	9.7	*18.2*	2.0	*1.6*
Microplacoid	17	3.0 – 5.2	*6.2 – 9.7*	4.1	*7.8*	0.6	*0.9*
Macroplacoid row	16	21.5 – 36.0	*51.8 – 59.5*	30.2	*55.9*	4.8	*2.3*
Placoid row	17	23.6 – 46.8	*68.5 – 75.8*	37.8	*71.6*	6.9	*2.4*
Claw 1 heights							
External primary branch	16	8.2 – 17.9	*21.9 – 30.7*	14.4	*27.4*	2.9	*2.4*
External secondary branch	16	5.5 – 14.8	*16.2 – 25.2*	10.9	*20.6*	2.3	*1.9*
Internal primary branch	16	8.4 – 16.1	*20.7 – 29.0*	13.2	*25.2*	2.5	*2.2*
Internal secondary branch	16	6.9 – 13.9	*17.1 – 24.1*	10.9	*20.8*	2.0	*2.1*
Claw 2 heights							
External primary branch	17	9.2 – 18.1	*22.7 – 30.3*	14.6	*27.6*	3.0	*2.7*
External secondary branch	16	7.2 – 15.5	*17.7 – 26.4*	11.9	*22.4*	2.6	*2.2*
Internal primary branch	17	8.0 – 16.2	*20.9 – 27.7*	13.3	*25.3*	2.3	*1.9*
Internal secondary branch	17	6.9 – 13.8	*19.0 – 23.6*	11.1	*21.1*	2.1	*1.3*
Claw 3 heights							
External primary branch	17	9.0 – 18.6	*20.4 – 31.9*	14.9	*28.3*	3.1	*3.2*
External secondary branch	17	7.1 – 14.4	*17.0 – 25.9*	11.7	*22.0*	2.5	*2.4*
Internal primary branch	17	8.5 – 18.3	*23.0 – 31.5*	14.2	*26.9*	2.7	*2.0*
Internal secondary branch	17	7.3 – 14.6	*17.9 – 24.9*	11.8	*22.4*	2.1	*1.7*
Claw 4 heights							
Anterior primary branch	17	10.2 – 19.2	*25.1 – 34.6*	15.9	*30.3*	2.5	*2.5*
Anterior secondary branch	17	6.6 – 14.6	*16.3 – 25.6*	11.9	*22.5*	2.3	*2.1*
Posterior primary branch	17	10.9 – 21.2	*27.1 – 36.2*	16.3	*31.1*	2.8	*2.6*
Posterior secondary branch	17	7.8 – 15.0	*20.4 – 27.8*	12.4	*23.5*	2.0	*1.9*

**Table 10.** Measurements [in μm] of selected morphological structures of eggs of *Paramacrobiotus fairbanksi* Schill, Förster, Dandekar & Wolf 2010 Canadian population mounted in Hoyer’s medium (N – number of specimens/structures measured, RANGE refers to the smallest and the largest structure among all measured eggs; SD – standard deviation).

CHARACTER	N	RANGE	MEAN	SD
Egg bare diameter	15	62.9 – 89.7	75.0	9.5
Egg full diameter	15	88.7 – 117.5	101.5	9.3
Process height	42	11.3 – 17.0	13.9	1.5
Process base width	42	13.6 – 18.9	15.9	1.2
Process base/height ratio	42	100% – 136%	115%	10%
Inter-process distance	41	1.0 – 5.5	2.7	1.0
Number of processes on the egg circumference	14	10 – 12	10.8	0.8

**Table 11.** Measurements [in μm] and pt values of selected morphological structures of individuals of *Paramacrobiotus fairbanksi* Schill, Förster, Dandekar & Wolf 2010 Madeira population mounted in Hoyer’s medium (N – number of specimens/structures measured; RANGE refers to the smallest and the largest structure among all measured specimens; SD – standard deviation, *pt* – ratio of the length of a given structure to the length of the buccal tube expressed as a percentage).

CHARACTER	N	RANGE		MEAN		SD	
		μm	*pt*	μm	*pt*	μm	*P^t^*
Body length	15	476 – 1036	–	714		174	
Buccopharyngeal tube							
Buccal tube length	15	49.4 – 69.3	–	59.3	–	7.2	–
Stylet support insertion point	15	37.9 – 53.8	*76.0 – 81.4*	46.4	*78.2*	5.6	*1.5*
Buccal tube external width	15	10.4 – 16.5	*21.0 – 25.9*	13.8	*23.2*	2.2	*1.6*
Buccal tube internal width	15	7.0 – 12.2	*14.1 – 19.1*	9.8	*16.4*	1.9	*1.5*
Ventral lamina length	13	29.5 – 39.6	*55.4 – 60.8*	34.7	*58.7*	3.7	*1.6*
Placoid lengths							
Macroplacoid 1	15	8.3 – 14.1	*16.7 – 21.8*	11.5	*19.3*	2.2	*1.6*
Macroplacoid 2	15	7.6 – 13.2	*14.9 – 19.1*	10.1	*16.9*	1.8	*1.3*
Macroplacoid 3	15	8.6 – 16.5	*16.7 – 24.0*	13.1	*21.8*	2.6	*2.1*
Microplacoid	15	3.2 – 5.9	*6.0 – 8.6*	4.1	*7.0*	0.8	*0.8*
Macroplacoid row	15	31.0 – 47.5	*60.1 – 71.7*	38.8	*65.2*	6.1	*2.9*
Placoid row	15	40.4 – 60.1	*78.8 – 86.8*	49.6	*83.4*	7.4	*2.8*
Claw 1 heights							
External primary branch	15	13.8 – 21.8	*27.9 – 31.9*	17.8	*30.0*	2.5	*1.5*
External secondary branch	15	9.5 – 15.7	*18.5 – 25.3*	12.9	*21.8*	1.7	*1.8*
Internal primary branch	15	13.4 – 19.6	*24.6 – 31.2*	16.3	*27.5*	2.2	*1.6*
Internal secondary branch	15	10.0 – 16.0	*18.0 – 24.7*	13.1	*22.1*	2.1	*1.8*
Claw 2 heights							
External primary branch	15	14.2 – 22.3	*28.4 – 32.7*	18.3	*30.8*	2.5	*1.3*
External secondary branch	15	11.8 – 17.4	*22.4 – 26.9*	14.6	*24.6*	1.9	*1.5*
Internal primary branch	15	13.9 – 20.4	*26.5 – 31.8*	16.9	*28.5*	2.3	*1.5*
Internal secondary branch	15	10.2 – 17.6	*20.6 – 25.8*	13.6	*22.9*	2.2	*1.7*
Claw 3 heights							
External primary branch	15	14.9 – 21.4	*24.4 – 32.9*	17.8	*30.1*	2.1	*2.2*
External secondary branch	15	11.6 – 16.5	*20.1 – 27.7*	14.3	*24.3*	1.7	*2.0*
Internal primary branch	15	14.0 – 21.2	*25.5 – 33.1*	17.3	*29.2*	2.5	*1.9*
Claw 4 heights							
Internal secondary branch	15	11.0 – 21.0	*21.0 – 30.7*	14.2	*23.8*	2.7	*2.3*
Anterior primary branch	15	16.7 – 23.2	*31.7 – 37.6*	20.1	*34.0*	2.4	*1.7*
Anterior secondary branch	15	12.2 – 18.6	*22.9 – 29.5*	15.5	*26.2*	1.8	*1.9*
Posterior primary branch	15	14.6 – 22.2	*28.8 – 35.5*	19.0	*32.1*	2.7	*1.6*
Posterior secondary branch	15	10.3 – 18.1	*20.4 – 26.2*	14.6	*24.5*	2.4	*1.5*

**Table 12.** Measurements [in μm] of selected morphological structures of eggs of *Paramacrobiotus fairbanksi* Schill, Förster, Dandekar & Wolf 2010 Madeira population mounted in Hoyer’s medium (N – number of specimens/structures measured, RANGE refers to the smallest and the largest structure among all measured eggs; SD – standard deviation).

CHARACTER	N	RANGE	MEAN	SD
Egg bare diameter	15	74.9 – 97.6	82.9	6.3
Egg full diameter	15	107.7 – 141.7	117.5	8.3
Process height	43	13.7 – 23.0	17.7	2.2
Process base width	43	15.1 – 23.8	18.7	2.0
Process base/height ratio	43	93% – 125%	106%	9%
Inter-process distance	44	3.0 – 6.2	4.6	0.7
Number of processes on the egg circumference	15	11 – 14	12.4	0.9

**Table 13.** Measurements [in μm] and pt values of selected morphological structures of individuals of *Paramacrobiotus fairbanksi* Schill, Förster, Dandekar & Wolf 2010 Mongolian population mounted in Hoyer’s medium (N – number of specimens/structures measured; RANGE refers to the smallest and the largest structure among all measured specimens; SD – standard deviation, *pt* – ratio of the length of a given structure to the length of the buccal tube expressed as a percentage).

CHARACTER	N	RANGE		MEAN		SD	
		μm	*pt*	μm	*pt*	μm	*pt*
Body length	14	553 – 864	–	717		104	
Buccopharyngeal tube							
Buccal tube length	14	53.7 – 67.6	–	61.5	–	3.6	–
Stylet support insertion point	13	40.7 – 51.2	*73.4 – 78.8*	46.9	*76.9*	2.8	*1.4*
Buccal tube external width	14	12.3 – 18.1	*20.1 – 26.8*	13.7	*22.2*	1.6	*1.9*
Buccal tube internal width	14	9.0 – 14.8	*14.3 – 21.9*	10.5	*17.0*	1.4	*1.7*
Ventral lamina length	11	36.0 – 43.1	*60.0 – 65.8*	39.0	*62.9*	2.2	*1.8*
Placoid lengths							
Macroplacoid 1	14	10.4 – 13.1	*16.2 – 21.8*	11.4	*18.6*	0.9	*1.5*
Macroplacoid 2	14	8.7 – 11.2	*14.1 – 18.8*	10.0	*16.3*	0.8	*1.4*
Macroplacoid 3	14	11.0 – 15.0	*17.5 – 25.0*	12.7	*20.8*	1.1	*1.8*
Microplacoid	14	4.6 – 7.1	*6.8 – 10.9*	5.6	*9.1*	0.7	*1.0*
Macroplacoid row	14	33.2 – 41.4	*56.6 – 64.9*	37.0	*60.2*	2.0	*2.5*
Placoid row	14	43.9 – 54.8	*74.1 – 84.6*	49.4	*80.5*	2.6	*3.1*
Claw 1 heights							
External primary branch	14	15.1 – 18.1	*23.7 – 30.1*	16.8	*27.3*	1.0	*1.9*
External secondary branch	14	11.7 – 15.2	*18.7 – 24.3*	13.6	*22.2*	1.1	*1.9*
Internal primary branch	14	14.8 – 18.2	*23.6 – 29.9*	16.5	*27.0*	1.1	*2.2*
Internal secondary branch	14	11.6 – 15.1	*18.7 – 24.4*	13.1	*21.3*	1.0	*1.9*
Claw 2 heights							
External primary branch	14	16.2 – 21.0	*26.2 – 35.1*	18.0	*29.4*	1.3	*2.5*
External secondary branch	14	12.6 – 16.9	*19.7 – 28.2*	14.6	*23.8*	1.3	*2.2*
Internal primary branch	14	13.0 – 19.6	*20.9 – 32.0*	15.9	*25.9*	2.1	*3.0*
Internal secondary branch	14	9.6 – 15.0	*15.5 – 24.6*	13.0	*21.2*	1.7	*2.3*
Claw 3 heights							
External primary branch	14	13.6 – 19.6	*21.8 – 31.5*	17.2	*28.0*	1.6	*2.8*
External secondary branch	14	10.7 – 16.4	*18.5 – 25.2*	13.4	*21.7*	1.7	*2.3*
Internal primary branch	14	13.6 – 18.0	*21.8 – 30.8*	16.5	*26.8*	1.4	*2.2*
Internal secondary branch	14	11.1 – 16.4	*17.9 – 25.1*	13.4	*21.8*	1.6	*2.3*
Claw 4 heights							
Anterior primary branch	14	15.6 – 21.9	*26.4 – 35.1*	18.6	*30.3*	1.7	*2.6*
Anterior secondary branch	14	10.7 – 17.1	*16.7 – 26.4*	14.3	*23.3*	1.7	*2.8*
Posterior primary branch	14	14.5 – 22.8	*23.6 – 34.5*	18.5	*30.1*	2.0	*2.9*
Posterior secondary branch	14	11.0 – 17.5	*18.5 – 28.5*	14.2	*23.1*	2.2	*3.3*

**Table 14.** Measurements [in μm] of selected morphological structures of eggs of *Paramacrobiotus fairbanksi* Schill, Förster, Dandekar & Wolf 2010 Mongolian population mounted in Hoyer’s medium (N – number of specimens/structures measured, RANGE refers to the smallest and the largest structure among all measured eggs; SD – standard deviation).

CHARACTER	N	RANGE	MEAN	SD
Egg bare diameter	6	64.0 – 83.5	73.0	8.0
Egg full diameter	6	87.4 – 112.4	96.8	9.5
Process height	16	11.0 – 16.9	14.2	1.9
Process base width	15	15.0 – 21.7	17.7	2.3
Process base/height ratio	15	114% – 137%	124%	7%
Inter-process distance	12	2.2 – 3.8	3.1	0.5
Number of processes on the egg circumference	5	11 – 15	12.3	1.9

## Data Availability

The datasets generated and/or analysed during the current study are available in the GenBank repository (all accession numbers listed in Table 2: ON911917-18, ON872386, ON872380-81, ON911919, ON872387, ON872382, ON911920-21, ON872388, ON872383, ON911922-;23, ON872389 and ON872384-85). The data of all sequences will be available for public access within a few days.
